# Multiple Micronutrients, Including Zinc, Selenium and Iron, Are Positively Associated with Anemia in New Zealand Aged Care Residents

**DOI:** 10.3390/nu13041072

**Published:** 2021-03-25

**Authors:** Sue O. MacDonell, Jody C. Miller, Michelle J. Harper, Malcolm R. Reid, Jillian J. Haszard, Rosalind S. Gibson, Lisa A. Houghton

**Affiliations:** 1 Department of General Practice and Primary Care, School of Population Health, University of Auckland, Auckland 1072, New Zealand; 2 Department of Human Nutrition, University of Otago, Dunedin 9054, New Zealand; jodym409@gmail.com (J.C.M.); michelle.harper@otago.ac.nz (M.J.H.); jill.haszard@otago.ac.nz (J.J.H.); Rosalind.gibson@otago.ac.nz (R.S.G.); lisa.houghton@otago.ac.nz (L.A.H.); 3 Trace Element Centre, Department of Chemistry, University of Otago, Dunedin 9054, New Zealand; malcolm.reid@otago.ac.nz

**Keywords:** anemia, inflammation, nursing home, anti-secretory medications, proton pump inhibitors, interleukin-6, soluble transferrin receptor, total body iron

## Abstract

Anemia is a significant comorbidity for older adults not fully attributable to iron deficiency. Low-grade inflammation and other micronutrient deficiencies also contribute. This cross-sectional study examined the relationships between nutrient and non-nutrient factors with hemoglobin and anemia in 285 residents (>65 years) of 16 New Zealand aged-care facilities. Blood samples were analyzed for hemoglobin, ferritin, sTfR, hepcidin, zinc, selenium, and interleukin-6 (IL-6), (with ferritin, sTfR, zinc and selenium adjusted for inflammation). Linear regression models examined the relationships between micronutrient biomarkers (iron, zinc, selenium, vitamin B-12 and D), age, sex, and health factors with hemoglobin. Thirty-two percent of participants exhibited anemia, although <2% had either depleted iron stores or iron deficiency. Plasma zinc and selenium deficiencies were present in 72% and 38% of participants, respectively. Plasma zinc and total body iron (TBI) were positively associated (*p* < 0.05) with hemoglobin, while gastric acid suppressing medications, hepcidin, and interleukin-6 were inversely associated. These relationships were maintained after the application of anemia cut-offs. These findings emphasize the importance of considering multiple micronutrient deficiencies as risk factors for anemia.

## 1. Introduction

Anemia in older adults is a significant comorbidity that increases with age and frailty [1]. Approximately 12% of community dwelling older adults over 65 years have hemoglobin concentrations indicative of anemia, and this proportion doubles for those aged over 80 years [2,3,4,5]. In aged-care facilities (nursing homes), the prevalence can be even higher, ranging from 25 to 60% [6,7,8,9,10,11,12]. Despite being frequently classified as mild and asymptomatic [8], anemia in older adults is associated with detrimental consequences that contribute to a reduced quality of life and include impairments in cognition, muscle strength and physical function as well as a greater incidence of frailty, admission to hospital and overall mortality [1,9,11,13,14].

The etiology of anemia in older adults is complex and attributed to multiple factors including age-associated physiological changes and chronic inflammation [1,15], medications [16,17] and micronutrient deficiencies, most commonly of iron and vitamin B-12 [1,18]. However, low hemoglobin concentrations have also been associated with deficiencies of other nutrients including vitamin D and the trace element selenium [19,20,21,22] and, in younger populations, with zinc [23].

Vitamin D deficiency has been associated with reduced hemoglobin concentrations in community dwelling older adults [24,25]. While there is limited evidence for an erythropoietic role of vitamin D [26], the anti-inflammatory effects of vitamin D may reduce the risk of anemia via the suppression of interleukin-6 (IL-6) and a subsequent upregulation of the iron-regulating peptide, hepcidin [18]. Vitamin D supplementation has also been observed to reduce circulating hepcidin concentrations in younger populations [27,28,29] likely due to the action of vitamin D on the hepcidin antimicrobial peptide (HAMP) gene [29].

An independent association between serum selenium and hemoglobin has been observed in adults aged over 65 years [19,20,30], while a similar relationship between plasma zinc and hemoglobin has also been identified, albeit in younger populations [23,31,32]. The relationships between hemoglobin and selenium, and hemoglobin and zinc have been attributed, at least in part, to the anti-oxidative protection of erythrocytes by the selenoprotein glutathione peroxidase [33] and the zinc-dependent enzyme, erythrocyte copper-zinc superoxide dismutase [34]. Furthermore, zinc may contribute to the maintenance of hemoglobin concentrations via the action of the zinc finger proteins GATA-1 and Gfi-1B that are involved in red blood cell production [35,36].

To our knowledge, associations between hemoglobin concentrations and the trace elements zinc and selenium have not been explored in the aged-care population. Earlier, we examined the influence of inflammation on biomarkers of iron, zinc, and selenium status in an aged-care population [37]. Here, we extend this research and explore potential associations between these micronutrients, vitamin B-12 and vitamin D, and non-nutrient factors with hemoglobin concentrations and anemia in New Zealand aged-care residents.

## 2. Materials and Methods

### 2.1. Participant Recruitment and Study Design

Details of recruitment and study design have been reported earlier [36]. Briefly, residents from sixteen aged-care facilities located throughout New Zealand were approached by trained research assistants to participate in this cross-sectional survey. Residents were considered eligible for inclusion if they were aged 65 years or older and had been resident at rest-home level for at least 12 weeks. Rest-home level care is the minimal level of care provided in New Zealand and is for individuals who require 24 h assistance with activities such as administering medications and showering. Generally, residents can self-feed and are mobile with supervision. Written, informed consent was obtained from 285 residents. For participants with cognitive impairment informed consent was provided by a named legal proxy. Data collection was conducted in two phases. In Phase One, participants were recruited from February to April (late summer/autumn) 2014, and Phase Two from July to September (late winter/spring) 2014. Ethical approval for the study was obtained from the University of Otago Human Ethics (Health) Committee (H13/118), and it is registered with the Australian New Zealand Clinical Trials Registry (ACTRN12617001575325).

### 2.2. Sociodemographic and Health Data

Clinical records, participants and aged-care facility staff provided medical history and demographic data. Anti-secretory medications known to suppress gastric acid secretion (proton pump inhibitors (PPIs) and H2 antagonists) [38], were identified from participants’ medical records and coded as categorical variables: prescribed or not prescribed. Current smoking status was determined from participants’ response to the question ‘Do you currently smoke cigarettes?’

### 2.3. Anthropometrics and Malnutrition

Anthropometric measurements were taken in triplicate by the same trained research staff using standardized techniques and calibrated equipment as described elsewhere [37]. Height was estimated from ulna length using published conversion charts [39]. Body Mass Index (BMI) was calculated and classified as non-obese (BMI < 30 kg/m^2^) or obese (BMI ≥ 30 kg/m^2^) [40].

Malnutrition risk was assessed using the Mini Nutritional Assessment Short Form (MNA-SF) and each participant subsequently classified as being of normal nutrition status, at risk of malnutrition, or malnourished [39].

### 2.4. Blood Collection and Processing

Early-morning fasting peripheral venous blood samples were drawn into clot-activator vacutainers (for hemoglobin, biomarkers of iron, vitamin B-12 and vitamin D), or K2-EDTA trace-element-free (BD#368381) vacutainers (Beckton Dickinson, Franklin Lakes, NJ, USA) for plasma zinc and selenium. A complete blood count was performed immediately by local laboratories, while remaining blood samples were chilled immediately and transported on the same day to the Human Nutrition Laboratory at the University of Otago where they were processed using rigorous trace-element free procedures [41]. Aliquots of serum and plasma were held frozen at −80 °C until required for further analysis.

### 2.5. Laboratory Analyses

Methods for the analysis of micronutrient status (full blood count, serum ferritin, soluble transferrin receptor (sTfR) and plasma zinc and selenium) and inflammatory biomarkers markers (high-sensitivity C-reactive protein (CRP), α-1-acid glycoprotein (AGP) and interleukin-6 (IL-6)) have been described previously [36]. Serum ferritin, serum sTfR, plasma zinc and plasma selenium concentrations were adjusted for the effect of subclinical inflammation using the regression method recommended by the BRINDA Project [42]. Details of the adjustment method have been reported elsewhere [37]. Briefly, individual micronutrient concentrations (serum ferritin, serum sTfR, plasma zinc and plasma selenium) were adjusted mathematically using *β*-coefficients from linear regression models where the unadjusted micronutrient biomarker was the dependent and IL-6 the independent variables. A reference IL-6 value was set to the maximum of the lowest decile to prevent over-adjustment of micronutrient biomarkers for participants with negligible inflammation [42].

The prevalence of micronutrient deficiency for each biomarker was calculated from values adjusted for the effect of inflammation and the following reference limits: plasma zinc concentration <10.7 μmol/L for women and <11.3 μmol/L for men [41]; selenium concentration <0.82 μmol/L [43]; and for depleted iron stores, serum ferritin concentration <15 μ g/mL [4,44]. The sTfR cut-off derived from the original ELISA method of Erhardt et al. (>8.3 mg/L) [45] was converted to account for the use of the Tina-quant assay (Roche Diagnostics GmbH) which yields values which are 30% lower [45]. This established a cut-off value >5.3 mg/L. Iron deficiency was also assessed using TBI calculated from the equation of Cook and colleagues [46] where values <0 mg/kg represent tissue iron deficiency [46,47].

Anemia was defined by the age and sex-specific criteria of Looker et al. [48]; for men hemoglobin concentrations <133 g/L (aged 65–69) or <124 g/L (aged 70 and older); and for women hemoglobin concentrations <120 g/L (aged 65–69) and <118 g/L (aged 70 and older). Iron deficiency anemia was defined as the presence of anemia combined with TBI < 0.0 mg/kg.

Serum hepcidin concentration was determined using commercially available enzyme-linked immunosorbent assay (ELISA) kits (Creative Diagnostics, Shirley, NY, USA). Results from the ELISA kits were determined from standard curves prepared from calibrations run simultaneously with the test samples. The inter-assay CV based on a pooled serum (*n* = 9) was 10.0%. Serum vitamin D concentrations (25-hydroxyergocalciferol and 25-hydroxycholecalciferol) were determined using isotope-dilution LC–tandem MS on an API 3200 instrument (Applied Biosystems, Foster City, CA, USA) based on the method of Maunsell et al. [49], and as described previously [50]. Vitamin D deficiency was defined as serum 25(OH)D concentrations <50 nmol/L [51,52].

Serum vitamin B-12 was measured by automated ECLIA (Elecsys 2010^®^; Roche Diagnostics GmbH, Mannheim, Germany). Means ± SD and CV for the two manufacturer-provided controls (Elecsys Preci-Control Varia 1 and 2) analyzed with each reagent kit were 472.37 ± 18.04 pg/mL; 3.8% and 1100.64 ± 40.74 pg/mL; 3.7%, respectively, and were within the range of the results provided by the manufacturer. Pooled serum inter-assay CV was 7.3% (*n* = 7). Vitamin B-12 concentrations <150.0 pm/L were considered deficient [53].

Serum creatinine concentrations were determined via a creatinine Jaffé Gen.2 kinetic colorimetric assay (Cobas c311^®^; Roche Diagnostics GmbH) with daily analysis of control material. Means were 96 ± 1.0 µmol/L (CV 1.0%) and 368 ± 5.8 µmol/L (CV 1.6%), respectively and fell within the expected ranges. Estimated glomerular filtration rate (eGFR), an indicator of renal function, was determined using the Chronic Kidney Disease Epidemiology Collaboration (CKD-EPI) online calculator [54] including variables serum creatinine, age, and sex.

### 2.6. Statistical Methods

The distributions of the biochemical variables were visually assessed and described using geometric means (95% CI) if right skewed. Participants who identified as Māori (*n* = 3) or other non-European ethnicities (*n* = 4) were not included in data analysis, as the proportion (<1.3%, respectively) of the total sample was not sufficient to support inference beyond a European sample. Participants with micronutrient biomarker concentrations above clinically feasible values were excluded from analysis because these likely reflected either an underlying clinical condition such as liver disease or haemochromatosis [41,55,56] (ferritin > 1100 μg/L (*n* = 3)) or sample contamination (zinc > 22 μmol/L (*n* = 2)).

Demographic and anthropometric differences between participants who were included or not included in analysis or who did not provide blood samples were assessed using the χ2 test for categorical variables except for current smoking status where the frequency was less than five participants. In this instance the Fisher’s exact test was used. Difference in continuous variables was assessed using the Wilcoxon rank-sum (Mann–Whitney) test.

Linear regression models were used to examine relationships with hemoglobin. Independent variables included in the models were: micronutrient biomarkers (iron, zinc, selenium, vitamin B-12 and D), health, demographic and biochemical factors known to be related to hemoglobin, specifically: age, sex [57], obesity [20], renal function (measured by eGFR) [9], malnutrition [1], inflammation (measured by serum CRP and IL-6) [1,9,15,20], current smoking status [58,59] and anti-secretory medications [16,17]. Biochemical factors were log-transformed if right-skewed. All factors were standardised so that estimates of association are presented in units of SD, rather than the differing units of the factor, thus allowing for comparison of the strength of association with hemoglobin. Initially, univariate regression models were generated, followed by a multivariate model that included all biomarkers and factors known to be related to hemoglobin (sex, obesity, malnutrition risk, CRP, renal function, and smoking status). Where variables provided information on the same parameter (e.g., iron status), the variable with the strongest R-squared was chosen to be included in the multivariate model. Residuals of regression models were plotted and visually assessed for homogeneity of variance and normality. All regression models applied clustered (aged-care facility) robust standard errors to account for potential correlations within individual facilities. Similar methods were applied to determine predictors of anemia but using logistic regression. Interactions between age and sex were investigated for all models.

All statistical analyses were performed using Stata statistical software package (version 16.1; Stata Corp, College Station, TX) and all tests were 2-sided with statistical significance determined by *p* < 0.05.

## 3. Results

Demographic, health, and micronutrient status for 285 participants provided blood samples and who self-identified as being of European ethnicity and are shown in Table 1. Demographic and health characteristics of those who provided a blood sample (*n* = 285) compared to those of European ethnicity who declined phlebotomy (*n* = 17) were not significantly different (*p* > 0.05). Almost half were malnourished or at risk of malnutrition (45.9%), and a similar proportion were prescribed anti-secretory medications. One or more of the three inflammatory biomarkers (CRP and/or AGP and/or IL-6) were elevated in 50% of participants (Table 1).

The prevalence of both depleted iron stores and iron deficiency (with or without the presence of anemia) was very low, although almost one third (31.6%) had anemia, of whom the majority (60.1%) had one or more raised inflammatory marker (Table 1). Only one participant had low serum vitamin B-12 and < 20% had serum 25(OH)D concentrations below 50 nmol/L. Conversely, the prevalence of plasma zinc and selenium deficiency was greater, at 71.9% and 38.3%, respectively.

### 3.1. Hemoglobin

Univariate regression revealed statistically significant associations between hemoglobin and age, anti-secretory medications, hepcidin, IL-6 and plasma zinc and selenium (*p* < 0.05) (Table 2). All biomarkers of iron status, (ferritin, sTfR and TBI) were also significantly associated with hemoglobin. Total body iron explained the greatest proportion of the variance (7.4%) compared to other biomarkers of iron status and as such, was used as the iron biomarker in the final adjusted model. Sex, obesity, malnutrition risk, CRP, renal function, and smoking status, while not statistically significant, were included in the final model due to the a priori selection criteria. The final adjusted model showed hemoglobin concentration was inversely associated with anti-secretory medications, serum IL-6 and serum hepcidin. Moreover, those taking anti-secretory medications had mean hemoglobin concentrations of 3.8 (95% CI −6.8, −0.7) g/L lower than those not taking these medications (*p* = 0.019). In contrast, hemoglobin was positively associated with TBI and plasma zinc (Table 2), with mean hemoglobin concentrations 4.9 (95% CI 2.2, 7.6) g/L higher for each SD rise in plasma zinc concentration (*p* = 0.002). Plasma selenium, while significantly associated with hemoglobin (mean difference (95% CI) = 3.8 (1.4, 6.1); *p* = 0.004) in the univariate model, was strongly attenuated in the final adjusted model (mean difference (95% CI) = 0.7 (−1.4, 2.7); *p* = 0.504).

No statistically significant interaction between age and sex was noted (*p* = 0.944). The final model explained 31% of the variance in hemoglobin and there was no evidence of collinearity among the covariates.

### 3.2. Anemia

For each 1 SD increase in plasma zinc, the odds of having anemia were approximately 53% lower (OR: 0.47; 95% CI: 0.30, 0.74; *p* = 0.001) (Table 3) while the odds ratio for anemia for those who were zinc deficient increasing almost five times that of zinc replete residents (OR (95% CI): 5.01 (1.75, 14.3)). Likewise, the odds of anemia declined by 36% for every 1 SD higher TBI. Conversely, the odds of being anemic increased for participants who were taking anti-secretory medication (OR: 1.81, 95% CI: 1.15, 2.86; *p* = 0.010) and who had higher concentrations of the inflammatory marker IL-6 (OR: 1.61, 95% CI: 1.17, 2.20; *p* = 0.003) and hepcidin (OR: 1.67, 95% CI: 1.07, 2.60; *p* = 0.023) (Table 3). No statistically significant association between anemia and current smoking status, plasma selenium or serum concentrations of vitamin B-12 or 25(OH)D were observed (*p* > 0.05).

## 4. Discussion

The present study reports low hemoglobin concentrations, indicative of anemia, in approximately one-third of a New Zealand European aged-care cohort. Iron deficiency was virtually absent; however, iron status was positively associated with hemoglobin and inversely associated with anemia. In contrast, there was a high prevalence of zinc and selenium deficiencies and lower plasma zinc levels were associated with greater odds of anemia. While lower selenium levels were also associated with greater odds of anemia, this was no longer the case after adjustment for other biomarkers, specifically plasma zinc.

The overall prevalence of anemia in the present study is similar to that observed previously in other aged-care populations [7,8,9,10], although markedly higher compared to community dwelling older adults in New Zealand [4,5] and elsewhere [2,3]. Frailty, polypharmacy, impaired renal function and the presence of multiple co-morbidities and underlying chronic inflammation have all been attributed to the higher prevalence of anemia in the aged-care setting [8,9,10,11,12]. Furthermore, anemia in this setting has been identified as a marker of increased mortality risk [11] which requires careful investigation to identify and manage underlying etiologies [61].

To our knowledge, this is the first study to observe a positive relationship of zinc on hemoglobin status in an elderly population, although it has been described previously in school-aged New Zealand children [23] and women of reproductive age [2,3,4]. In these populations, including our aged-care cohort, zinc deficiency was more prevalent than iron deficiency. The high prevalence of zinc deficiency is likely to be associated with both dietary factors and age-related physiological changes in zinc metabolism. Poor appetite, leading to reductions in food intake, including low intakes of red meat, are known to characterize inadequate zinc intakes among elderly New Zealanders [62]. Furthermore, recent analyses of Canadian aged-care menus demonstrated sufficient provision of dietary iron, while 50% of residents had zinc intakes below the estimated average requirement [63]. Host-related factors that result in poor absorption, excessive losses, or impaired utilization of zinc may also be attributable to the suboptimal zinc status observed here. These factors include a possible decrease in zinc absorption with aging [64], potentially exacerbated by hypochlorhydria, enteric infection, and/or malabsorption syndromes [41].

In the present study, the risk of anemia for those who were zinc deficient was almost five times that of zinc replete participants (OR (95% CI): 5.01 (1.75, 14.3)), emphasizing that adequate iron status alone does not ensure protection against anemia. Zinc may contribute to the maintenance of hemoglobin concentrations via multiple mechanisms. Such mechanisms may include the action of the zinc finger proteins GATA-1 [35] and Gfi-1 B [36] on erythroid differentiation and development; while the zinc-dependent enzyme 5′-aminolevulinic acid (ALA) dehydratase is involved in the synthesis of porphyrin, a key component of hemoglobin [65]. Furthermore, zinc deficiency has been shown to reduce the integrity of red blood cell membranes by making them susceptible to osmotic stress, thereby increasing their fragility [34,66].

The attenuation of the significant positive association between selenium and hemoglobin in our cohort, is in contrast with the positive associations reported elsewhere in both community dwelling [19,20,22] and aged-care elderly [19]. However, adjustment for additional micronutrient biomarkers (zinc and vitamin D) in the present study may explain the divergent conclusions.

We found no significant association between vitamin D and hemoglobin concentrations in this aged-care population. This finding is supported by the conclusions of a recent systematic review and meta-analysis that found no significant effect of vitamin D supplementation on hemoglobin concentrations. There was, however, a small positive effect of vitamin D supplementation on transferrin saturation concentrations (mean difference: 1.54; 95% CI: 0.31, 2.76; *p* = 0.01) [67]. High dose vitamin D supplementation has previously been shown to reduce the concentration of both hepcidin and inflammatory markers [27], which are inversely associated with the anemia of inflammation. The contrasting findings of these and other vitamin D studies may be influenced by variations in the type, dose, and duration of vitamin D supplementation. Further work which investigates the impact of vitamin D on the complete range of iron homeostasis markers is, therefore, warranted, especially in older adults where the etiology of anemia can be multifactorial. Our findings that IL-6 and hepcidin were associated with low hemoglobin concentrations support the hypothesized mechanism for the anemia of inflammation (AI) that is commonly observed in older adults [2]. IL-6 appears to induce the expression of hepcidin, which affects iron metabolism and is considered the major mediator of AI. Furthermore, an additional contribution by hepcidin to AI through impaired erythropoiesis has also been proposed [68] and may account for the observed association of IL-6 and hepcidin with lower hemoglobin concentrations.

Anti-secretory medications which suppress the production of gastric acid were a stronger determinant of both hemoglobin and anemia in our population compared to the selected micronutrients. Nearly half of the cohort were prescribed these medications—a level that is substantially higher than those previously published [69]. The potential mechanism of how these medications are associated with anemia is based on the hypothesis that suppression of gastric acid production leads to an increase in pH. This change in pH prevents the reduction in iron salts and proteolysis of vitamin B-12, leading to reduced bioavailability and subsequent deficiency of these micronutrients [16,17,38,70]. However, the low prevalence of iron and vitamin B-12 deficiency in our cohort does not lend support to this proposed mechanistic relationship between anti-secretory medications and hemoglobin.

Nonetheless, the relationship between anti-secretory medications and hematological markers is conflicting. Our findings are consistent with Sarzynski et al. showing an inverse trend in hemoglobin concentrations with proton pump inhibitor therapy [16]; two large randomized clinical trials of PPI therapy, however, found no clinically significant change in either serum hemoglobin or vitamin B-12 concentrations [17]. These inconsistencies are further supported by recent reviews and practice guidelines that acknowledge only a slight risk of iron or vitamin B-12 deficiency secondary to PPIs [70,71] and as such, monitoring for micronutrient deficiencies is not warranted [71]. The observed effect of these medications on hemoglobin concentrations may instead be related to the chronic inflammatory states that initiate prescription of anti-secretory medications, thereby making the medications an indicator of anemia risk, rather than being directly causal.

The strengths of this study include the detailed assessment of multiple micronutrient biomarkers which were adjusted for the effect of both overt and sub-clinical inflammation to ensure a more accurate representation of status [42]. It should also be noted that while plasma zinc and selenium are considered suitable markers for the assessment of population status, they do not provide a reliable indication of the trace element status of an individual. [41,72]. A full dietary and clinical assessment should be used to investigate any suspected zinc or selenium deficiency. Furthermore, the cross-sectional design of this study does not elucidate a causal effect of zinc deficiency in lower hemoglobin concentrations or anemia. It is also important to note that participants were recruited from the least dependent level of aged care in New Zealand which must be considered before extending these results to older adults in other care settings.

## 5. Conclusions

Our results extend the extensive body of research regarding anemia in older adults by showing that suboptimal zinc status in the absence of iron deficiency is a significant risk factor for low hemoglobin and anemia. This finding emphasizes the importance of considering multiple micronutrients when interpreting markers of anemia. Micronutrient intervention studies in this age group should consider and measure inflammatory status to better assist our understanding of the complex mechanisms which arise from the interplay of inflammation, hepcidin and micronutrient status to detrimentally affect hemoglobin and anemia.

In summary, anemia is a complex clinical condition that is highly prevalent in the aged-care setting. Appropriate management requires careful evaluation of factors beyond iron deficiency. The effect on hemoglobin status of inflammation, additional micronutrient deficiencies, and impaired renal function should be fully investigated before confirming a treatment plan.

## Figures and Tables

**Table 1:** Demographic, health, and selected micronutrient data for 285 New Zealand aged care residents ^1^.

Variable	*n*	Geometric Mean (95% CIs) ^2^
Age, years (±SD)	285	85.0 ± 7.5
Sex (male), *n* (%)		92 (32.3)
Obese ^3^, *n* (%)	279	55 (19.7)
Smoking status, *n* (%)	276	
Non-smoker		266 (96.4)
Current smoker		10 (3.6)
Malnutrition ^4^, *n* (%)	279	
Normal nutrition status		151 (54.12)
At risk of malnutrition		109 (39.1)
Malnourished		19 (6.8)
Gastric acid supressing medication ^5^, *n* (%)	284	135 (47.5)
Serum ferritin ^6^, μg/L	282	94.1 (84.4, 104.8)
Depleted iron stores (serum ferritin <15 μg/L), *n* (%)		6 (2.0)
Serum sTfR ^6^, mg/L	283	3.2 (3.0, 3.3)
sTfR > 5.3 mg/L, *n* (%)		22 (7.7)
Total body iron ^7^, mg/kg	277	7.7 (7.1, 8.4)
Total body iron < 0 mg/kg, *n* (%)		4 (1.3)
Hemoglobin, g/L	282	125.4 (123.6, 127.2)
Anemia ^8^, *n* (%)		89 (31.6)
Iron deficiency anemia ^9^, *n* (%)		4 (1.3)
Hepcidin, ng/mL	284	7.9 (7.4, 8.3)
Plasma zinc ^6^, μmol/L	281	10.0 (9.8, 10.1)
Low plasma zinc ^10^, *n* (%)		202 (71.9)
Plasma selenium ^6^, μmol/L	282	0.88 (0.85, 0.91)
Low plasma selenium (<0.82 μmol/L), *n* (%)		108 (38.3)
Serum 25(OH)D, nmol/L	285	75.4 (69.2, 82.2)
Low serum 25(OH)D (<50 nmol/L), *n* (%)		49 (17.2)
Serum vitamin B-12 pg/mL	285	424.8 (399.2, 452.1)
Low serum vitamin B-12 (<150 pg/mL), *n* (%)		1 (0.4)
Serum CRP, mg/L	281	3.7 (3.3, 4.2)
Elevated serum CRP (>5 mg/L), *n* (%)		97 (34.5)
Serum AGP, g/L	283	0.84 (0.81, 0.87)
Elevated serum AGP (> 1 g/L), *n* (%)		77 (27.2)
Serum Interleukin-6, pg/mL	285	5.8 (5.2, 6.4)
Elevated serum IL-6 concentration ^11^, *n* (%)		278 (97.6)
eGFR (mL/min/1.73 m^2^)	283	57.8 (55.4, 60.3)

^1^ Data are presented for participants who provided a blood sample and self-identified ethnicity as NZ European and Other Ethnicity. ^2^ Biochemical variables are presented as geometric means (95% CIs) unless noted as frequency (percent). ^3^ BMI ≥ 30 kg/m^2^. ^4^ Coded by the Mini Nutritional Assessment Short Form categories [39]. ^5^ Prescribed anti-secretory medications (proton pump inhibitors or H_2_ receptor antagonists). ^6^ Adjusted to remove the effects of subclinical inflammation by regression with IL-6. ^7^ Total body iron = −[log10(sTfR × 100 O/ferritin) − 2.8229]/0.1207 where ferritin is adjusted for inflammation and sTfR is adjusted for inflammation and converted to values representative of the ELISA method of Flowers [45]. ^8^ Hemoglobin <133 g/L for men aged 65–69, <124 g/L for men aged 70 and older, <120 g/L for women aged 65–69 and <118 g/L for women aged 70 and older [48]. ^9^ Anemia and total body iron < 0.0 mg/L. ^10^ Adjusted plasma zinc <10.7 μmol/L for women and <11.3 μmol/L for men [41]. ^11^ Il-6 greater than the age and sex specific median [60]. AGP α-1 glycoprotein; CRP, C-reactive protein; eGFR, estimated glomerular filtration rate; sTfR, soluble transferrin receptor.

**Table 2:** Association of demographic, health, and biochemical factors with serum hemoglobin (g/L) among New Zealand aged-care residents of European ethnicity ^1^.

Variable	*n*	Univariate Regression		Final Adjusted Model ^1^	
		*β*-Coefficient (95% CI)	*p*	*β*-Coefficient (95% CI)	*p*
Age, years	282	−0.25 (−0.50, −0.01)	0.048	0.03 (−0.22, 0.29)	0.776
Female	282	−2.93 (−6.82, 0.95)	0.128	−2.55 (−6.42, 1.32)	0.181
Gastric acid supressing medications *(yes)*	281	−7.83 (−11.77, −3.90)	0.001	−3.75 (−6.81, −0.70)	0.019
Current Smoker	273	−1.95 (−12.47, 8.57)	0.698	0.00 (−8.06, 8.06)	>0.999
Obesity ^2^	276	−3.25 (−7.71, 1.21)	0.141	−2.60 (−6.33, 1.13)	0.158
Malnutrition ^3^	276		0.077		0.176
At risk of malnutrition compared to normal nutrition status		−2.11 (−4.37, 0.16)		0.10 (−3.33, 3.53)	
Malnourished compared to normal nutrition status		−5.07 (−13.03, 2.90)		−4.15 (−9.27, 0.96)	
Serum ferritin ^4,5^	279	3.74 (2.20, 5.27)	<0.001		
Serum sTfR ^4,5^	281	−2.56 (−4.93, −0.19)	0.036		
Total Body Iron ^4,5^	278	4.02 (2.47, 5.58)	<0.001	2.44 (0.13, 4.75)	0.040
Plasma zinc ^4,5^	278	5.37 (2.19, 8.56)	0.003	4.89 (2.17, 7.60)	0.002
Plasma selenium ^4,5^	279	3.77 (1.40, 6.14)	0.004	0.67 (−1.41, 2.74)	0.504
Serum CRP ^5^	279	−1.74 (−4.21, 0.74)	0.155	0.67 (−2.07, 3.41)	0.609
Serum IL-6 ^5^	282	−2.96 (−4.16, −1.75)	<0.001	−3.00 (−4.76, −1.25)	0.002
Serum hepcidin ^5^	281	−3.34 (−5.83, −0.84)	0.012	−2.01 (−4.00, −0.02)	0.048
Serum vitamin D ^5^	282	−0.03 (−2.06, 2.00)	0.975	0.62 (−1.83, 3.07)	0.596
Serum vitamin B-12 ^5^	282	−0.10 (−2.76, 2.55)	0.934	−0.21 (−2.19, 1.77)	0.825
eGFR ^5^	281	2.06 (−0.30, 4.41)	0.082	−0.09 (−2.67, 2.48)	0.939

^1^*R^2^* of the adjusted model = 0·307; constant (95% CI) is 128 (107, 148); *p* < 0.001, *n* = 254. ^2^ coded as non-obese if body mass index < 30 kg/m^2^ and obese if body mass index ≥ 30 kg/m^2^, ^3^ coded by Mini Nutritional Assessment Short Form categories [39], ^4^ adjusted to remove the effects of subclinical inflammation using the BRINDA regression method and IL-6 [37], ^5^ serum ferritin, serum soluble transferrin receptor, serum B-12, serum hepcidin, serum CRP, serum AGP, and serum IL-6 all log-transformed; after log-transformation (if any) the variable was standardised so units are standard deviations, AGP α-1 glycoprotein; CRP, C-reactive protein; eGFR, estimated glomerular filtration rate; IL-6, interleukin-6; sTfR, soluble transferrin receptor.

**Table 3:** Logistic regression for anemia in 278 New Zealand aged-care residents of European ethnicity ^1,2^**.**

Variable	Univariate Regression		Final Adjusted Model	
	OR (95% CI)	*p*	OR (95% CI)	*p*
Age, years	1.02 (0.97, 1.07)	0.427	0.98 (0.91, 1.05)	0.515
Sex (female)	0.73 (0.40, 1.31)	0.293	0.68 (0.31, 1.52)	0.349
Current smoker	1.55 (0.33, 7.18)	0.576	2.68 (0.28, 25.72)	0.392
Malnutrition ^3^		0.009		0.137
At risk of malnutrition (compared to normal nutrition status)	1.82 (1.24, 2.67)		1.79 (0.91, 3.53)	0.092
Malnourished (compared to normal nutrition status)	1.29 (0.43, 3.85)		1.21 (0.38, 3.80)	0.747
Obesity (BMI ≥ 30 kg/m^2^)	1.69 (1.13, 2.53)	0.011	1.33 (0.69, 2.59)	0.394
Gastric acid supressing medications (yes)	3.07 (1.90, 4.95)	<0.001	1.81 (1.15, 2.86)	0.010
Serum ferritin ^4,5^	0.61 (0.50, 0.73)	<0.001		
Serum sTfR ^4,5^	1.28 (0.94, 1.74)	0.113		
Total Body Iron ^4,5^	0.60 (0.48, 0.74)	<0.001	0.64 (0.45, 0.92)	0.017
Plasma zinc ^4,5^	0.46 (0.29, 0.72)	0.001	0.47 (0.30, 0.74)	0.001
Plasma selenium ^4,5^	0.57 (0.39, 0.84)	0.005	0.92 (0.59, 1.45)	0.731
Serum CRP ^5^	1.37 (0.997, 1.89)	0.052	0.97 (0.63, 1.50)	0.892
Serum IL-6 ^5^	1.65 (1.40, 1.95)	<0.001	1.61 (1.17, 2.20)	0.003
Serum vitamin D ^5^	0.86 (0.67, 1.12)	0.261	0.81 (0.55, 1.18)	0.273
Serum vitamin B-12 ^5^	0.94 (0.67, 1.31)	0.703	0.89 (0.64, 1.23)	0.473
Serum hepcidin ^5^	1.96 (1.44, 2.67)	<0.001	1.67 (1.07, 2.60)	0.023
eGFR ^5^	0.80 (0.62, 1.02)	0.076	1.01 (0.73, 1.40)	0.958

^1^ Pseudo R^2^ of the adjusted model = 0·244, ^2^ anemia defined as hemoglobin <133 g/L for men aged 65–69, <124 g/L for men aged 70 and older, <120 g/L for women aged 65–69 and <118 g/L for women aged 70 and older [48], ^3^ coded by Mini Nutritional Assessment Short Form categories [39]. ^4^ Adjusted to remove the effects of subclinical inflammation using BRINDA regression method [37]. ^5^ Serum ferritin, serum soluble transferrin receptor, serum B-12, serum hepcidin, serum CRP, serum AGP, and serum IL-6 all log-transformed; after log-transformation (if any) the variable was standardized so units are standard deviations. BMI, body mass index; BRINDA, Biomarkers Reflecting Inflammation and Nutritional Determinants of Anemia; CI, confidence interval; CRP, C-reactive protein; eGFR, estimated glomerular filtration rate; OR, odds ratio; sTfR, soluble transferrin receptor.

## Data Availability

The data presented in this study may be available on request from the corresponding author. The data are not publicly available due to privacy and ethical requirements.
